# Development of Biodegradable Bio-Based Composite for Bone Tissue Engineering: Synthesis, Characterization and In Vitro Biocompatible Evaluation

**DOI:** 10.3390/polym13213611

**Published:** 2021-10-20

**Authors:** Muhammad Umar Aslam Khan, Saiful Izwan Abd Razak, Mohamed Nainar Mohamed Ansari, Razauden Mohamed Zulkifli, Nurliyana Ahmad Zawawi, Muhammad Arshad

**Affiliations:** 1 BioInspired Device and Tissue Engineering Research Group, School of Biomedical Engineering and Health Sciences, Faculty of Engineering, Universiti Teknologi Malaysia, Skudai 81300, Johor, Malaysia; 2 Department of Biosciences, Faculty of Science, Universiti Teknologi Malaysia, Skudai 81300, Johor, Malaysia; razauden@utm.my (R.M.Z.); nurliyana@utm.my (N.A.Z.); 3 National Center for Physics, Nanosciences and Technology Department, Quaid-i-Azam University Islamabad Campus, Islamabad 44000, Pakistan; Arshad_pr2002@yahoo.com; 4 Centre of Advanced Composite Materials, Faculty of Engineering, Universiti Teknologi Malaysia, Skudai 81300, Johor, Malaysia; 5 Institute of Power Engineering, Universiti Tenaga Nasional, Kajang 43000, Selangor, Malaysia

**Keywords:** biopolymer, biomaterials, biodegradations, polysaccharide, bone tissue engineering

## Abstract

Several significant advancements in the field of bone regenerative medicine have been made in recent years. However, therapeutic options, such as bone grafts, have several drawbacks. There is a need to develop an adequate bone substitute. As a result, significant bone defects/injuries pose a severe challenge for orthopaedic and reconstructive bone tissue. We synthesized polymeric composite material from arabinoxylan (ARX), β-glucan (BG), nano-hydroxyapatite (nHAp), graphene oxide (GO), acrylic acid (AAc) through free radical polymerization and porous scaffold fabricated using the freeze-drying technique. These fabricated porous scaffolds were then coated with chitosan solution to enhance their biological activities. The complex structure of BG, nHAp, GO was studied through various characterization and biological assays. The structural, morphological, wetting and mechanical analyses were determined using FT-IR, XRD, XPS, SEM/EXD, water contact angle and UTM. The swelling (aqueous and PBS media) and degradation (PBS media) observed their behavior in contact with body fluid. The biological activities were conducted against mouse pre-osteoblast cell lines. The result found that BGH3 has desirable morphological, structural with optimum swelling, degradation, and mechanical behavior. It was also found to be cytocompatible against MC3T3-E1 cell lines. The obtained results confirmed that the fabricated polymeric scaffolds would be a potential bone substitute to regenerate defective bone with different loading bearing applications for bone tissue engineering.

## 1. Introduction

Bone is a connective tissue supporting and protecting the rest of the body systems and organs by storing bone marrow, minerals and ions. Bone constantly remodels to adapt to the mechanical environments and heal the minor lesions [1]. However, certain bone defects of critical dimensions may cause delayed and harming bone function restoration. Bone-related impairments and diseases are approximately half of all chronic diseases in people 50 years above, a big challenge in medical science [2]. Over the last century, a wide range of activities, strategies, and surgical materials were used to study to regenerate defective bone tissue. Bone grafts are used in bone tissue application to restore function and aesthetics that were damaged due to bone defects. To mimic the bone tissue to be regenerated, the bone substitute’s physicochemical and biomechanical properties should be recognized, including architecture, pore size distribution, biocompatibility, degradability, osteoconduction osteoinductivity and osteogenesis. Biomaterials are categorized based on their source (allogeneic, alloplastic, autogenous and xenogeneic, etc.), stoichiometry composition (ceramics and bioglass and polymers, etc.), or interaction with biological systems [3,4]. Sizeable segmental bone defects cannot be repaired by themselves, even though bones can heal and/or regenerate. The “gold standard” for repairing bone defects is still autologous bone grafting. Autologous bone grafting is affected by several issues (i.e., secondary damage, high donor site morbidity, minimal shape, autogenous bone insufficiency, etc.). Because of these flaws, it is not widely used in the clinical system [5].

A biodegradable scaffold is placed as a temporary implant into the impaired sites of defective bones to support and activate bone regeneration which is slowly degraded and replaced by the new bone in bone tissue engineering. Tissue engineering scaffolds were developed and tested using bioactive ceramics and polymers with similar chemical cosmetics of a natural bone, supporting osteogenesis and bonds with the host tissue [6,7]. Bioceramics, despite their beneficial biochemical activity, are brittle and biodegrade slowly, limiting their medical applications. In contrast, biopolymers offer several advantages over ceramics. Their mechanical and biodegradation features can be optimized significantly for some applications. These are especially well-suited to implant in the body and known for easy term formation into desired orientations and shapes [8,9]. Little mechanical hardness and shape retention breakdown are the two most severe issues with polymer scaffolds.

For use as scaffolds, many types of natural and synthetics polymers were investigated. The scaffolds used in tissue repairing are bone, tendon, cartilage, skeletal muscle, skin, ligament, vascular tissues, and neural tissues. Natural polymers, which are comprised of proteins (such as collagen, silk and fibrin, etc.) or polysaccharides (including chitosan, alginate, starch, and arabinoxylan, etc.), have restricted mechanical characteristics, immunogenic prospect, and demand [10,11,12]. Poly(glycolic acid) (PGA), poly(L-lactic acid) (PLLA), and their copolymers, such as poly(DL-lactic-co-glycolic acid), are some of the most common synthetic polymers (PLGA). However, these synthetic polymers prevent cellular growth in a 3D structure due to poor cell adhesion and hydrophobic surfaces [13]. They are also deficient in specific functional groups, which could be used to alter the surface further. Previous synthetic biopolymers implanted in vivo discharged acidic degradation products and triggered a long-term immune response harmful to the host tissues. Furthermore, during polymer erosion, some specific bulk hydrolyzing PLGA copolymers were observed to considerably diminish the osteogenesis of the regenerating bone [14].

Polysaccharides are the most common type of carbohydrate in foods. These are polymeric carbohydrates made up of monosaccharide units linked by glycosidic linkages that form long chains. Chitosan is a polysaccharide cationic biopolymer that belongs to the polysaccharide class of biodegradable biopolymers. It is non-toxic, biodegradable, and non-antigenic. Unlike synthetic polymers, chitosan has a hydrophobic nature that encourages cell adhesion, differentiation, and proliferation while causing only a minor inflammatory response when implanted [15]. Chitosan scaffolds are osteoconductive, meaning they can promote bone regeneration in vitro and in vivo. Despite its widespread recognition as a biocompatible compound, chitosan is mechanically fragile and unsound, and swelling causes it to lose its predetermined form for transplantation [16]. A natural polymer-based system has yet to be developed with a sufficiently porous scaffold with appropriate mechanical and biological properties comparable to or better than chitosan [17]. β-Glucans (beta-glucans) is a naturally occurring biopolymer composed of β-D-glucose polysaccharides. It is primarily found in bacteria, cereals, and fungi cell walls, and its physicochemical properties vary a lot depending on the source [18]. β-Glucans usually have a linear backbone with 1–3-glycosidic bonds. However, these differ in viscosity, solubility, molecular mass, branching structure, and gelation properties, resulting in many physiological effects [19].

Here we report a biodegradable porous composite scaffold, its synthesis and properties. Briefly, polymeric composites were synthesized using the free radical method from arabinoxylan (ARX), β-glucans (BG), nano-hydroxyapatite (nHAp), graphene oxide (GO), acrylic acid (AAc) and freeze-drying technique was used to fabricate porous scaffolds. These fabricated scaffolds were coated with chitosan to enhance their biological activities. X-ray diffraction (XRD), Fourier-transform infrared spectroscopy (FT-IR), scanning electron microscope (SEM), water contact angle, and universal testing machine (UTM) were used to characterize the structural, morphological, wetting analyses, and mechanical testing of the fabricated scaffolds, respectively. The swelling (aqueous and PBS media) and degradation analysis (PBS media) were conducted to investigate their behavior. The biological activities were determined against mouse pre-osteoblast (MC3T3-E1) cell lines.

## 2. Materials and Methods

### 2.1. Materials

Β-glucan is a natural polymer extracted from barley flour and supplied by DSP Gokyo Food and Chemical Co., Ltd., Osaka, Japan. Acrylic acid (monomer), N, N’-methylene bisacrylamide (crosslinker) and graphene oxide (CAS# 763713-1G) were supplied by Sigma Aldrich Selangor, Malaysia. Hydroxyapatite nanoparticles (<100 nm particle size, >95%), phosphate buffer saline (PBS) solution and ethanol were supplied by Sigma-Aldrich, Selangor, Malaysia. All chemicals and reagents were used as received.

The osteoblast cells were purchased from the American Type Culture Collection (ATCC), Manassas, VA, USA. ThermoFisher Scientific (Waltham, MA, USA) supplied Alpha-MEM (α-MEM). L-glutamine penicillin/streptomycin and fetal bovine serum (FBS) were provided by Hyclone Laboratories Inc. (Logan, UT, USA).

### 2.2. Methods

#### 2.2.1. Extraction and Purification of β-Glucan

β-glucan was extracted using a standard method with a slight modification [20]. Briefly, 100 g fine barley flour was dispersed into 500 mL deionized water and pH = 10 of the slurry was maintained by Na_2_CO_3_ (20%, *v*/*w*). For 30 min the suspension was stirred at 45 °C and centrifuged at 18,000× *g* for 20 min (4 °C and pH 4.5) to isolate protein contents. The precipitated proteins were removed, and equal amounts of absolute ethanol were added to the suspension with slow stirring to precipitate β-G. Then, the suspension was centrifuged (4000× *g*) for 15 min to get β-G precipitates and kept at 4 °C overnight. These were freeze-dried to have fine powder of β-G and packed in an airtight glass jar.

#### 2.2.2. Extraction and Purification of Arabinoxylan

The psyllium (Plantago ovata) seed husk extracted arabinoxylan with slight modification by a well-reported method by Saghir et al. [21]. Briefly, the dust and stones were removed from psyllium seed husk (500 g) and put into deionized water (3 L) overnight. The swelled psyllium seed husk was then blended with NaOH soln. (2.5%) and stirring for 5 min. The insoluble seed husk was separated using a muslin cloth to get the pure gel. Then, it was coagulated by concentrated acetic acid. The resultant gel was neutralized using deionized water and freeze-dried to obtain a well-dried powder of arabinoxylan (ARX).

#### 2.2.3. Polymeric Composite Synthesis

The polymeric composite materials were synthesized by free radical polymerization. Briefly, biopolymeric powder (ARX = 1 g and BG = 1 g) was dispersed in deionized water (25 mL) to have homogenized suspension. Different amounts of nHAp (1.4, 1.5 and 1.6) were dispersed in deionized water (10 mL) to have homogenized suspension by sonication. These all suspensions were transferred into a round bottom flask (two-necked) and stirred for 45 min. Then, GO (0.3 mg) was added to the reaction media and stirred for 30 min at 65 °C. The amount of GO was optimized in our previous studies. After, 65 °C, the monomer (AAc = 0.50 mL) and crosslinker (N,N-MBA = 0.05% of AAc) were added into well homogenized mixture. The free-radical polymerization reaction was initiated by adding potassium persulphate (0.05 g) and heated for 3 h at the same temperature under an inert environment with constant stirring. The reaction was stopped heating, and nitrogen gas was removed. The reaction media was cooled and vacuum filtered, and the residue was washed 3 times with deionized water to ensure the unreacted species were from the product. The synthesized polymeric composite was dried in the oven at 50 °C overnight and packed for further use, and the actual yield was found to be approximately 63.82%. The composition of materials is mentioned in Table 1. The codes were assigned after a different amount of nHAp. Each sample (2 g) dispersed into deionized water (5 mL) to make a homogenized slurry and shifted into aluminium moulds (h = 2 cm and d = 1 cm). These moulds were frozen at −40 °C for 24 h and lyophilized by freeze-drying methodology to have porous and crack-less well-dried scaffolds.

## 3. Characterizations

### 3.1. Fourier-Transform Infrared Spectroscopy (FTIR)

The structural properties and chemical interactions were determined of fabricated scaffolds using FTIR (Shimadzu FTIR-8100A, Tokyo, Japan). The analysis was conducted using ATR mode, and 4000–400 cm^−1^ was scanning range. The average scan was 150 with a resolution of 4.0 cm^−1^.

### 3.2. X-ray Diffraction (XRD)

The diffraction pattern of fabricated scaffolds was determined by X-ray diffractometry. The analysis was conducted using XRD (Bruker AXS D8 (Kontich, Belgium)) using CuKα radiation (λ = 1.540 Å) using the voltage at 30 kV with a current of 30 mA. The XRD spectrum was obtained with a fixed range of 10 to 60°. The scan speed was 1.5 min^−1^ and 0.03 o step size.

### 3.3. Scanning Electron Microscope (SEM)/Energy Dispersive X-ray (EDX)

The morphological analysis of fabricated scaffolds was conducted by SEM (JEOL-JSM 6480, Peabody, MA, USA). The SEM was coupled with EDS to analyze elemental composition. Before analysis, the fabricated scaffolds were gold-sputtered.

### 3.4. X-ray Photoelectron Spectroscopy (XPS)

The XPS analysis was performed by a Scienta-Omicron system coupled with a micro-focused monochromatic Al K-alpha X-ray source. The detailed survey scan analysis was performed by operating X-ray at 15 KeV with 700-micron spot size. The constant analyzer energy (CAE) was kept at 100 eV. The charging effects were avoided by applying a combined low energy/ion flood source. The Matrix software was used to perform data acquisition, and Igor pro was applied to analyze the XPS fitting procedure. The shrilly background was corrected, and Gaussian–Lorentzian line shape was used to study the Curve fitting of detailed spectra. The C1s were used as reference data by fixing binding energy at 284.8 eV.

### 3.5. Mechanical Testing

The mechanical testing was performed using a universal testing machine (UTM, Testometrics, Rochdale, UK) to obtain strain–stress curves. The strain–each fabricated cylindrical scaffold determined stress curve with dimensions 1.5 cm (width) and 1.7 cm (height) at 0.5 mm/min speed.

### 3.6. Wetting

The hydrophilicity/hydrophobicity was determined of fabricated scaffolds through wetting analysis. The contact angle meter (XCA-50) (VCA-Optima, AST Inc., Tacoma, WA, USA) determines the wetting behavior of fabricated scaffolds. The wetting was investigated at a different time interval to analyze the effect of time.

### 3.7. Swelling and Degradation Analysis

The swelling of well-dried fabricated scaffolds was determined in aqueous and PBS media at 37 °C. The fabricated samples of each scaffold were weighted (50 mg) and placed in corresponding media to determine swelling behavior. The samples were taken out after a specific interval, and the excess surface liquid was removed carefully by tissue paper to record actual weight. The scaffold sample was placed into media until equilibrium, and the swelling percentage was determined by Equation (1).

(1) $$Swelling\;\left(\%\right)=\frac{W_{s}-W_{d}}{W_{d}}\times 100$$

The degradation studies of fabricated scaffolds were observed in PBS media (pH 7.4 at 37 °C) for 30 days. The fabricated scaffold was weighed (50 mg) carefully and placed in PBS media. The scaffold samples were taken out after a specific time interval. The scaffolds were rinsed with deionized water and oven-dried weight. The percentage degradation was determined by Equation (2).

(2) $$Degradation\;\left(\%\right)=\frac{W_{i}-W_{t}}{W_{t}}\times 100$$

whereas: *W_t_* = scaffold weight at “*t*” time, “*W_i_*” initial scaffold weight.

### 3.8. In Vitro Biological Studies

#### 3.8.1. Cell Morphology

The cell viability of the polymeric composite was studied against MC3T3-E1 cell lines in triplicates. The 24-well plate was coated by a fine layer of polymeric scaffold and sterilized using a UV light for 1 h. The cell lines were maintained in culture media (1% penicillin/streptomycin, 1% (2 mM) L-glutamine, 10% FBS and α-MEM. The cell lines (5000 cell/cm^2^) were seeded in a 24-well plate triplicate by cell culture media. The 24-well plates were incubated for 24, 48 and 72 h under in vitro conditions, i.e., 37 °C, 5% CO_2_ and 90% humidity. After removing cell culture media, the grown cells were fixed by absolute ethanol for 5 min at room temperature. The cell was gold-sputtered and SEM (JEOL-JSM 6480, Peabody, MA, USA) to capture the photographs at different intervals of time.

#### 3.8.2. Cell Viability and Optical Density

The MC3T3-E1 cell lines were used to determine the scaffolds’ cell viability and optical density by taking gelatin (0.1%) as +ive control, and 1% dimethyl sulfoxide (DMSO) was taken −ive control. These well plates were incubated at different time intervals (24, 48, and 72 h) under in vitro conditions (5% CO_2_ at 37 °C with 90% humidity), as mentioned in our previous work [22]. The culture well plates were again incubated with neutral red (40 µg/mL) media for 2 h, Repetto et al. [23]. The excess NR stain was removed by washing cells with PBS solution for 20 min. These cells were destained by dye-staining solution (50% deionized water, 49% absolute ethanol and 1% glacial acetic acid) for 10 min. An absorbance microplate reader (ELx-800) (BioTek, Winooski, VT, USA) was used at 570 nm to record optical density. A fluorescence microscope (Nikon ECLIPS TS100) with a 488 nm excitation filter was used to study cell morphology. The background interference of the microscopy was fixed by Vital dye (fluorescein diacetate (FDA)), created by coated scaffolds. Equation (3) was used to calculate cell viability percentage.

(3) $$Cell\;viability=\frac{OD_{s}}{OD_{c}}\times 100$$

where *OD_s_* = sample optical density and *OD_c_* = control optical density.

### 3.9. Statistical Analysis

The statistical analysis was conducted by software (IBM SPSS Statistics 21) of the obtained data and presented in mean, standard error (S.E.) and have shown as Y-error bars in figures. (*n* = 3), and *p* < 0.05.

## 4. Results and Discussion

### 4.1. FTIR Analysis

**Figure 1:**
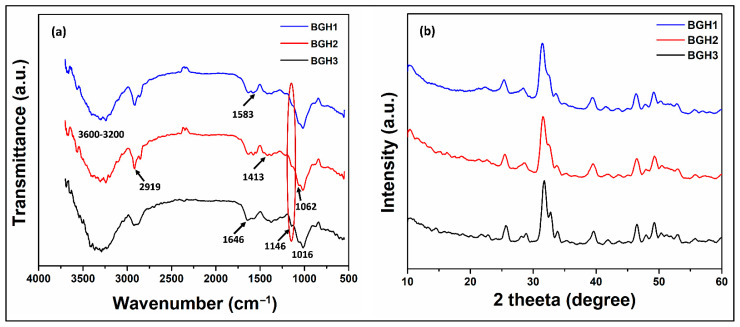
(**a**) FTIR spectra and (**b**) XRD pattern of porous scaffolds samples.

The structural analysis of the porous scaffolds was analyzed by FTIR spectral profile as shown in Figure 1a. The vibration peaks at 571, 608 cm^−1^ and 1089 cm^−1^ present the triply degenerated P−O stretching and O−P−O bending (first two) of HAp. The characteristic bands from 560 to 600 cm^−1^ and 1000–1100 cm^−1^ exhibit moiety of calcium phosphate due to nHAp [22]. It was further noted that increasing amount of nHAp cause the appearance of 1146 and 1062 cm^−1^, which are belonged to stretching vibrations of –C–O–C– and –C–O–, respectively, which is a clear indication that increasing nHAp amount decrease polymerization contents due to increased ceramic contents and decreased monomeric contents [24]. However, the –OH group confirmed HAp in the scaffold at 630 cm^−1^. The band from 1750 to 1600 cm^−1^ is attributed to the C–O stretching of GO, NN-MBA and free hydroxyl (–OH) [25]. The peak at 2928 cm^−1^ is typical of saturated aliphatic C–H stretching vibrations, and broadband at 3100–3500 cm^−1^ is attributed to inter and intra-hydrogen bonding due to biopolymers (ARX, BG) [6]. The broadband also confirms the ceramic and other contents were linked via hydrogen bonding. These peaks and functional groups confirm the available interaction (covalent bonding, hydrogen and van der Waal forces, etc.) synthesis of the polymeric composite material via free-radical polymerization. Hence, the development of our desired composite scaffolds was demonstrated by the presence of these vibration peaks and bands.

### 4.2. XRD Analysis

XRD analysis was conducted in the current research to observe the crystalline and phase analysis of inorganic content in polymeric scaffolds, as shown in Figure 1b. XRD pattern of polymeric scaffolds was compared by standard JCPDS (PDF-4-932) data for HAp. The peaks at 25.89, 31.86, 32.59, 39.77, 47.57 and 49.33 o have corresponding plans such as (201), (217), (300), (310), (222) and (213), and these are the characteristic peaks of hydroxyapatite. A sharp peak was observed for HAp with good crystallinity. The characteristic peak of HAp with the highest intensity was found at a 2θ value of 31.86° with corresponding planes (211) and indicated a highly crystalline form of HAp [6]. The XRD pattern of the polymeric matrix has peaked for nHAp, AAc, GO, BG and ARX. The polymeric and ceramic contents have retained their crystalline phase in polymeric scaffolds, confirming the substantial change in their crystal structure. It also indicates the uniform and fine distribution of HAp in the polymeric matrix. It is analogous to the natural bone in which HAp is homogeneously dispersed into a collagen matrix [26]. The week and distinguish peaks at 21.78, 23.81 and 26.34 represented characteristic peaks of the polymeric matrix that establish HAp incorporation in polymeric scaffolds. The dispersion of HAp in polymeric solution and more significant diffraction of HAp nanoparticles have improved the crystalline phase and intensity of polymeric scaffolds. However, characteristic peaks of the polymeric matrix were observed in FTIR spectra. The entire composite scaffold exhibited an enhanced sharpness and intensities of characteristic HAp peaks. The increase in crystal sizes of HAp particles when introduced into polymeric scaffold could be likened to these reasons [27]. The decrease in crystalline behavior of BG-co-AAc was observed in composite polymeric materials with poor peaks for BG, GO and AAc. It can be due to the formation of covalent bonding, hydrogen bonding and other weak interaction during free-radical polymerization. These were developed in the synthesis of polymeric composite materials that imprints HAp into the polymeric matrix.

### 4.3. XPS Analysis

**Figure 2:**
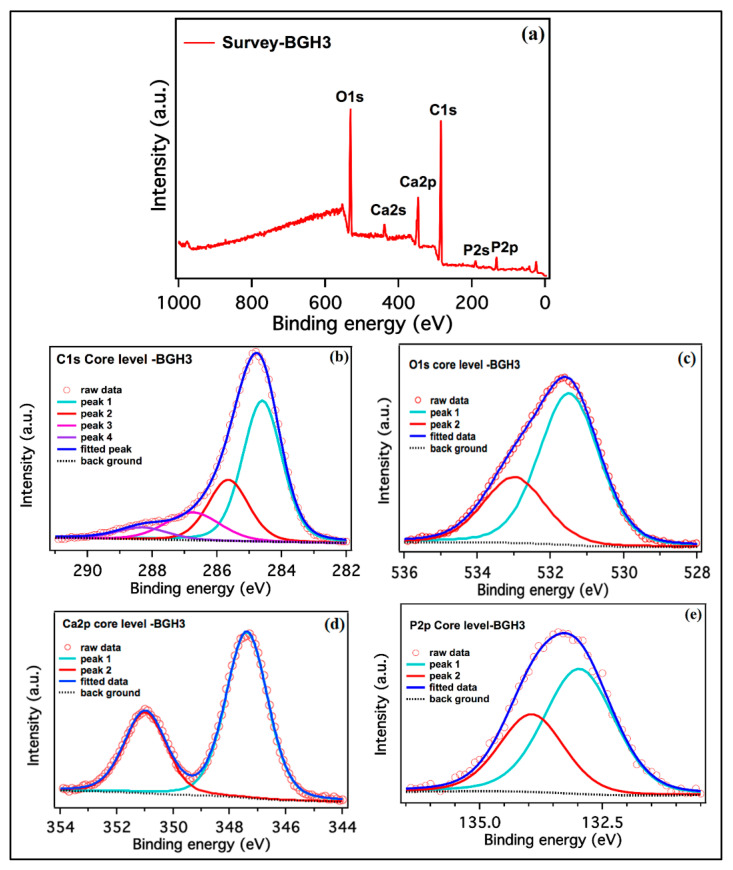
XPS spectra of porous scaffolds. (**a**) BGH-3 survey, (**b**) carbon, (**c**) oxygen, (**d**) calcium and (**e**) phosphorous high-resolution scans, respectively.

The XPS spectra collected from the as-synthesized BGH-3 sample is shown in Figure 2. The survey scan collected from BGH-3 shown in Figure 2a identified the carbon, oxygen, calcium, and phosphorus as we expected. The high-resolution scans of C1s, O1s, Ca2p and P2p, are shown in Figure 2b–e. The relative atomic percentages of all elements present on the surface of the sample and the binding energies position of all deconvoluted peaks are given in Table 2. The deconvoluted peaks of C1s spectra confirm sp2 bonded carbon (graphene) and functional groups [28]. Ca2p and P2p peaks correspond to calcium phosphate, and these peak assignments are also reported in the literature [29]. The first peak of O1s confirms the oxide of Ca, and other peaks are related to OH species [29]. Hence, the available different binding energy level confirms the different interaction during the synthesis of polymeric composite material via free radical polymerization.

### 4.4. Morphological Analysis

**Figure 3:**
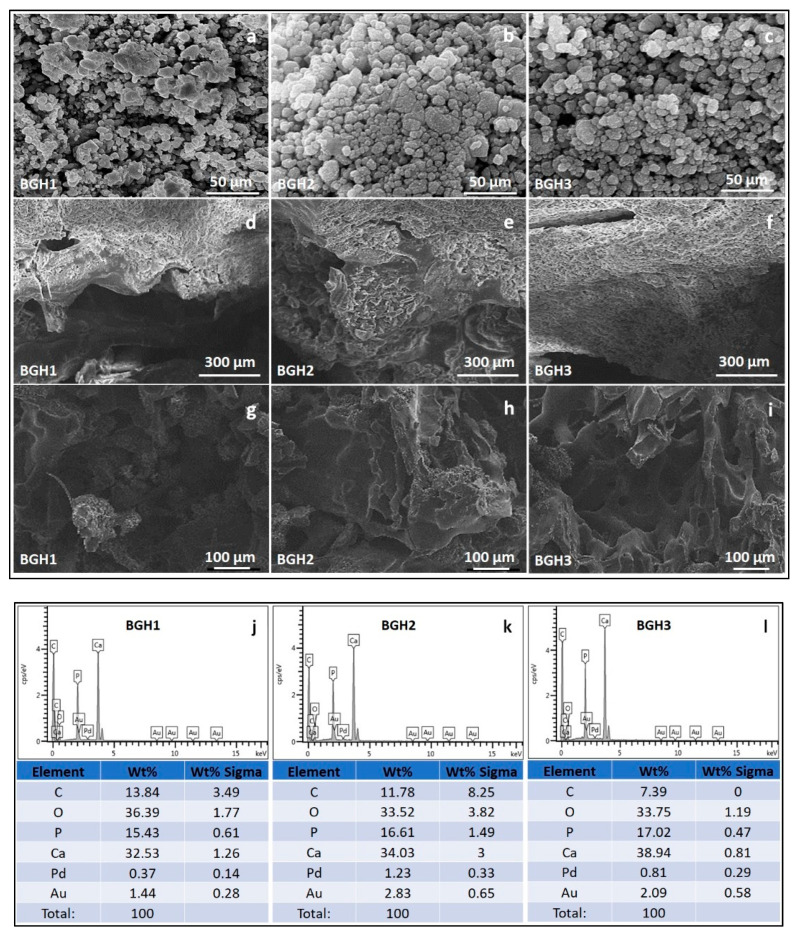
SEM morphologies of composite materials (**a**–**c**), scaffolds’ surface morphology at different scales (300 (**d**–**f**) and 100 (**g**–**i**) µm) and elemental analysis (**j**–**l**) by EDX.

The surface and porous morphologies are significant characters of any biomaterial for biomedical applications, especially bone tissue engineering. The rough surface and interconnected porosity are essential to proliferate, differentiate, and adhere to bone tissue support. These samples exhibit rough surface morphology, and it is also observed that increasing HAp amount caused interconnected porosity with regulated pore size, as shown in Figure 3. It is assumed as the polymer concentration increased as ceramic amount decreased, the viscosity of the solution increased, preventing it from flowing out, causing some pores to partially close.

The presence of adequate porosity in the scaffold is essential to regulate critical processes such as nutrient delivery to cells, metabolite dispersion, pH stability, and cell signalling. In the early stages, the size of the pores is a significant factor for evaluating the closeness of cells. The later stages of tissue growth allow for three-dimensional cell-cell communication and space for cells to organize themselves in three dimensions. Because cell seeding in the scaffold’s centre and feeding the scaffolds’ inner surfaces are limited, smaller pores have their constraints. On the other hand, larger pores affect the scaffold’s stability and offer physical support for the cell seeding. Pores of sufficient size allow cells to migrate or adhere to a material’s surface [30]. Table 1 shows that the porosity of all scaffolds was greater than 55%. It indicates that they have substantial porosity and are effective for cell adherence and proliferation. Researchers found that increasing the porosity of macroporous biphasic calcium phosphate ceramic samples improved bone growth, implying the importance of high porous scaffolds for better cell growth in the scaffold [31]. The porosity of polymeric scaffolds BGH1, BGH2 and BGH3 are 54.69, 71.24, and 82.96%, respectively. The different porosity (%) of the fabricated scaffolds confirm that increasing HAp amount have effect over porosity (%) that also affect the different interaction (covalent, hydrogen bonding and van der Waal forces, etc.) during free radical polymerization. Pore size is a critical factor to consider when designing a tissue engineering scaffold. In terms of attachment, matrix deposition, and differentiation, it has an impact on cellular activity. Within the field of bone tissue engineering, there appears to be widespread agreement that the optimal pore size for bone tissue scaffolds is between 100 and 500 micrometres for better cell adhesion and proliferation, as well as vascular ingrowth [32]. The EDX analysis confirms the increase in HAp intensity as HAp amount increases, and no foreign component is found that causes any toxicity.

### 4.5. Mechanical and Porosity

Porous scaffold must mimic the native extracellular matrix (ECM) with desirable mechanical behavior and porosity, an essential requirement for bone tissue regeneration. The mechanical properties of the scaffolds should be similar to those of native bone tissue. The mechanical strength can be determined by the impact resistance of polymeric scaffolds to retain structural integrity during implantation [33]. Multiple aspects of a biomaterial, including topography, particle and crystal size, chemical nature, and pore size distribution, can significantly affect the cellular behavior for biological efficiency. Almost all of these features are linked to the compositions of ceramic and polymeric components during synthesizing the composite materials [34].

**Figure 4:**
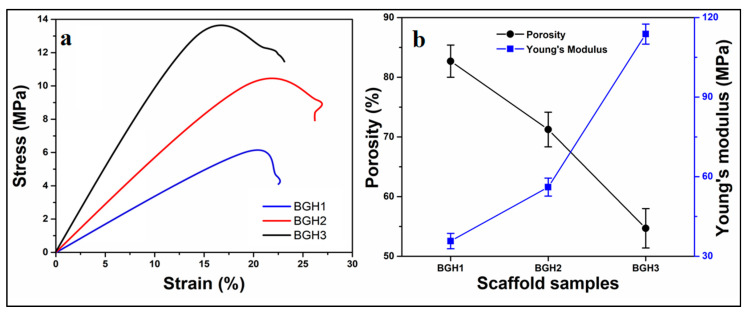
(**a**) Mechanical analysis via strain–stress curves, (**b**) relationship between porosity and Young’s modulus relationship.

The polymeric scaffolds’ machinal properties were conducted via stress-strain curves, as shown in Figure 4a. Since GO and HAp are well-known materials to enhance the mechanical and structural integrity of materials. The amount of GO was optimized, and different nHAp amounts were used to enhance the mechanical behavior of fabricated scaffolds [35]. Hence, the increasing amount of nHAp has increased compression strength (minimum = 6.19 and maximum = 13.76 MPa) and Young’s modulus (maximum = 113.75 and minimum = 35.73 MPa). It was noted that increased nHAp amount caused increased mechanical strength, but a decreased porosity was observed. The detailed properties of mechanical and porosity are given in Table 1. Since nHAp is a ceramic material and has several available active sites. The increasing nHAp caused increased mechanical strength due to crosslinking behavior that caused the close packing of polymeric scaffolds that reduced the porosity [15]. The relationship between porosity and Young’s modulus of the polymeric scaffolds have shown in Figure 4b. The variable nHAp amount has changed the chemical structure by affecting interface and matrix grains. Optimized amount of GO and increased HAp amount enhanced mechanical properties due to grain size boundary. The nHAp provides a vast surface area that interacts with polymeric matrix and other materials to regulate the porosity of polymeric scaffolds [36]. The porosity, mechanical strength and Young’s modulus has been summarized in Table 3.

### 4.6. Wetting Analysis

**Figure 5:**
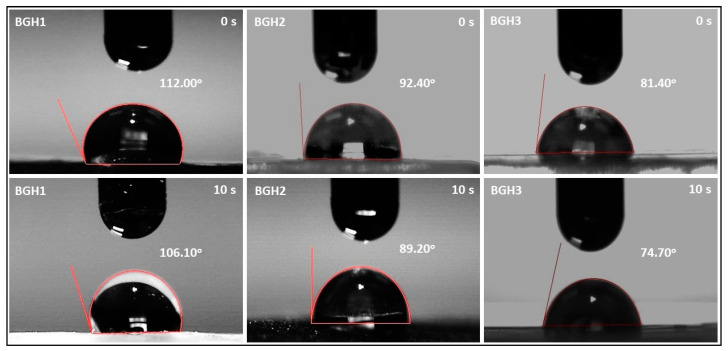
Wetting behavior of polymeric scaffolds via water contact angle.

**Figure 6:**
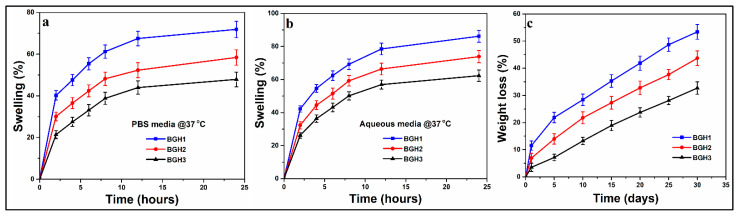
(**a**,**b**) swelling behavior of polymeric scaffold in different media (aqueous and PBS) and (**c**) in vitro biodegradation of polymeric scaffolds in PBS media.

Wetting of material surfaces is a crucial feature for tissue engineering, and it has an impact on cell-material interactions. Wetting of polymeric material is a vital property to achieve a suitable biological response. Wetting measurements are an essential part of the scientific analysis of biomaterial features. Contact angle measurements are one of the most common methods for determining the wetting behavior of polymeric biomaterial surfaces [37]. The hydrophilic surface is better for osteoblast growth and mineral deposition than the hydrophobic surface. The bioactivity and cytocompatibility of the scaffold are both critical factors and can be evaluated by the wetting behavior of scaffolds [38]. Figure 5 presents the wetting behavior concerning the time and quantity of nHAp. It was observed that an increasing amount of nHAp shifts hydrophobic behavior from hydrophilic. It is also worth mentioning that increasing water contact time shift hydrophobicity behavior towards hydrophilicity, as shown in Figure 6. Since these scaffolds possessed different wetting behavior due to different times. Hence, the scaffolds are hydrophilic, which have a water contact angle of less than 90°. This analysis indicates that the hydrophobicity can be modified by introducing hydrophilic materials and increasing water contact time with the polymeric scaffolds. The wetting behavior of the fabricated scaffolds has been summarized in Table 3.

### 4.7. Swelling and Degradation Analysis

The ability of biomaterials to swell is a crucial feature that facilitates the interaction of the biological system with biomaterials. It also regulates the sustained release of therapeutic agents. Swelling is a characteristic of polymeric materials that occurs when a solvent penetrates the void space of the polymeric chain network. The swelling causes the polymeric materials to expand. In some cases, external triggers (such as pH, ionic strength, temperature, etc.) can influence the swelling behavior of polymeric materials [39]. The polymeric content of the biomaterial is higher. It allows more fluids to exchange with the environment. Due to the large surface areas of the polymeric composite, it responds faster to exhibit swelling capacity than conventional polymeric composites [40].

Scaffold swelling behavior and structural integrity are critical for tissue engineering clinical use. In biological fluids, mostly biopolymers swell quickly. Initial swelling is desirable in the in vitro culture studies because the resulting increase in pore size enhances 3D cell adherence and proliferation. On the other hand, persistent swelling would result in a loss of mechanical integrity that develops compressive stress in the surrounding tissue [41]. The implantation site’s pH value and media condition significantly impact the swelling behavior of a scaffold. Because of the different HAp nanoparticles amounts, the swelling behavior of these fabricated scaffolds varies. The scaffolds swelled more in deionized water and less in PBS solution, as shown in Figure 6a. The data show that increasing nHAp in the scaffolds reduced swelling in deionized water and PBS media at 37 °C, as presented in Figure 6b. Because there was less nHAp and more polymeric content in BGH-3, it swelled the least (deionized water = 62.30%, PBS media = 47.87%). Due to the highest content of nHAp, BGH-1 had the maximum swelling (deionized water = 86.20%, PBS media = 71.90%). The interesting feature where HAp nanoparticles behave like a physical crosslinker is responsible for different swelling characteristics of scaffolds. Increasing the quantity of HAp nanoparticles in a polymeric material may cause additional crosslinking, reducing the material’s elasticity behavior [42]. 

The essential characteristics of polymeric composites for biomedical applications are degradability and biocompatibility. Polymeric composites are generally biocompatible and low in toxicity. As a result, they do not cause any unwanted biological responses at the molecular, cellular, or organ level. They contain more water, and the hydrophilic functional groups in the polymeric network are more numerous [43]. Because the polymeric substrates are arranged via various weak interactions, most polymeric composite-based drug delivery systems prepared by self-assembly are degradable. The degradation of scaffolds in PBS solution at 37 °C was evaluated, and degradation (%) was estimated by weight loss, as shown in Figure 6c. As the time spent immersed in the PBS solution increased, so did the weight loss. Because these are physical crosslinkers, increasing the amount of HAp nanoparticles has the reverse impact on degradation. The scaffold sample BGH1 showed the most degradation, while BGH3 showed the least. The crosslinking ability of the HAp nanoparticles is responsible for the different degradation properties of hybrid composite scaffolds [44]. It would be simple to conclude that increased HAp amount causes the scaffolds to crosslink more, resulting in a more compact structure that is less easily eroded. Increased crosslinking also changes the scaffold’s hydrophobicity to a more hydrophobicity (Figure 6c). The swelling and biodegradation behavior of the fabricated scaffolds has been summarized in Table 4.

### 4.8. In Vitro Studies

#### 4.8.1. Cell Morphology

**Figure 7:**
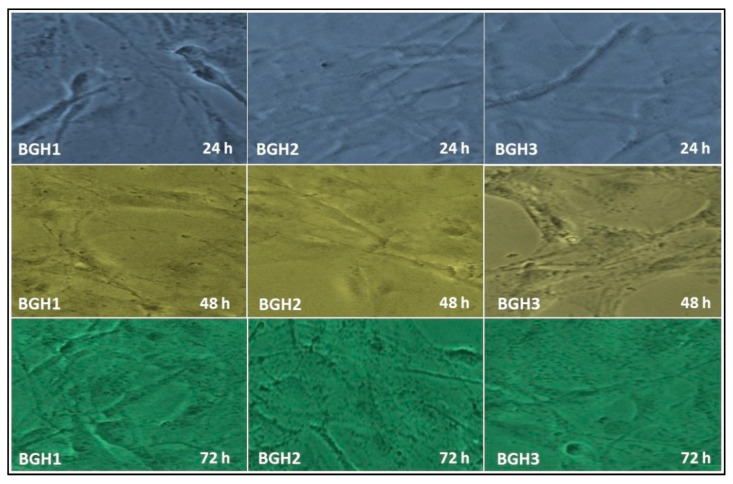
In vitro cell morphology of MC3T3-E1 against polymeric scaffolds (BGH1, BGH2 and BGH3) after different time intervals (24, 48, and 72 h).

**Figure 8:**
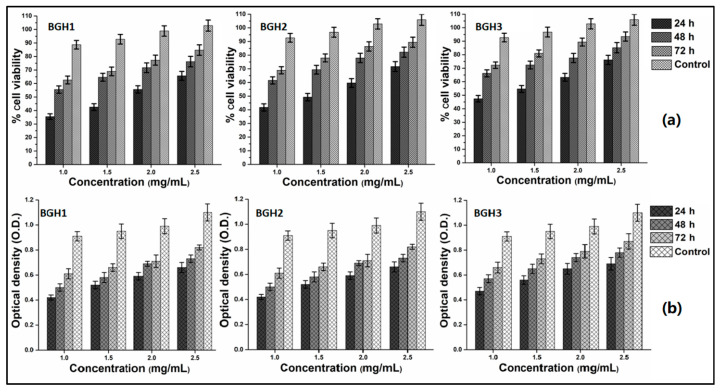
In vitro biocompatibility behavior of polymeric scaffolds against MC3T3-E1 at different concentrations (1, 1.5, 2 and 2.5 mg/mL) after different time intervals (24, 48 and 72 h). (**a**) cell viability and (**b**) optical density.

The cell adherence and morphology of the polymeric scaffold were evaluated against MC3T3-E1 cell lines, as shown in Figure 7. These polymeric scaffolds are multifunctional due to several available functional groups (–OH, –OPO_3_, –CH_3_ and –COOH, etc.) of GO, HAp, and the polymeric matrix. This multifunctionality encourages cell adherence that supports cell viability and proliferation due to hydrogen bonding [6]. It was observed that increasing HAp enhances active sites that are helpful for integrin bonding with the surface of polymeric scaffolds and improve cell adherence, differentiation with a proper cylindrical shape. The increased hydrophilicity with desirable multifunctionality helped material-DNA communication, and increased nHAp encouraged osteogenesis to heal host bone [6]. The rough and porous morphology of polymeric scaffolds has a microstructural surface that develops a feasible microenvironment that helps cell adherence and cell spreading in a cylindrical shape, as shown in Figure 8. Hence all polymeric scaffolds have proper cylindrical and stretched morphology, and mature cell adherence and morphology were observed after 72 h for BGH3.

#### 4.8.2. Cell Viability and Optical Density

The biological activities of polymeric were performed against MC3T3-E1 cell lines by in vitro analyses to determine cell viability (Figure 8a) assay and optical density (Figure 8b). The advancements in biomolecular science techniques have made it possible to collect osteoblast-precursor cells from various sources. The most common sources are bone marrow, umbilical blood, facial bones, subcutaneous fat, muscular tissue, osseous tissue, orthodontic pulp, and periosteum [45]. To study the concentration effect, the cell viability and optical density were performed using different concentrations (1, 1.5, 2 and 2.5 mg/mL). These polymeric scaffolds were incubated against MC3T3-E1 cell lines under in vitro analysis at different time intervals (24, 48 and 72 h). The cell viability, adherence and proliferation were increased with increasing HAp amount and maximum cell viability and proliferation after 72 h with mature cell adherence. The cylindrical cell shape was observed with proper adherence, and mature adherence was found after 72 h for BGH3. The increasing HAp amount with fixed GO amount increases the active sites that encourage cell adherence, which leads to cell viability and optical density, causing cell proliferation. Since the active sites (HAp) and oxygen-based (GO and BG) have several functional groups that improve microstructural behavior and micro-environmental for cell adherence that facilitate cell viability and proliferation as HAp is well known for osteogenesis [16]. The combined effect of BG, GO and HAp support cytocompatibility due to enhanced physicochemical properties. Hence, the polymeric scaffolds were biocompatible with improved cell viability and proliferation with mature cell adherence.

## 5. Conclusions

Excellent translational biomaterials demonstrate a ton of potential performance in the lab but have limitations in commercialization due to cast and other research aspects. Here we reported the synthesis of polymeric composite materials via the free radical method to fabricate polymeric scaffolds using the freeze-drying technique. The results demonstrate that the fabricated scaffolds have porous morphology, adjustable wetting, swelling, degradation and mechanical testing. In vitro bioactivities were performed against MC3T3-E1 at different times and concentrations. It was found that increasing ceramic (nHAp) amount enhances physicochemical and biomechanical characteristics. BGH3 was the best polymeric scaffold due to high mechanical (Young’s modulus and compression) with appropriate porosity and surface morphology due to maximum HAp amount with fixed GO quantity. The increasing HAp amount enhances the cytocompatibility and biocompatibility of cell adherence, leading to cell viability and proliferation due to the highly interrelated porous scaffold structure. It was found from the results that these polymeric scaffolds have desirable structural, morphological and biocompatible properties with different load-bearing mechanical behaviors. These polymeric scaffolds would be promising biomaterials to regenerate fractured bone for bone tissue engineering applications.

## Figures and Tables

**Table 1:** Chemical composition of biomaterial to fabricate scaffolds.

Sample Name	ARX (g)	BG (g)	AAc (g)	GO (mg)	nHAp (g)
BGC1	1	1	0.05	0.03	1.4
BGC2	1	1	0.05	0.03	1.5
BGC3	1	1	0.05	0.03	1.6

**Table 2:** The peak position and relative atomic% of the scaffold samples.

Elements	Peaks	Deconvoluted Peak Positions (eV)	Spin-Orbit Splitting	Bonding	Relative Atomic%
Carbon (C1s)	peak 1	284.6 ± 0.05	--	C=C (sp2 carbon)	62.90
	peak 2	285.4 ± 0.05	--	C–O	
	peak 3	287 ± 0.05	--	C≥O	
	peak 4	288.5 ± 0.05	--	O–C=O	
Oxygen (O1s)	peak 1	531.1 ± 0.05	--	O–P–O	25.89
	peak 2	532.51 ± 0.05	--	–OH	
Calcium (Ca2p)	peak 1	347.39 ± 0.05	2P_3/2_	CaO	
	peak 2	350.97 ± 0.05	2P_1/2_	CaCO3	6.09
Phosphorous (P2p)	peak 1	133 ± 0.05	2P_3/2_	O–P–O	5.12
	peak 2	133.89 ± 0.05	2P_1/2_	P–O	

**Table 3:** Summaries the mechanical and porosity of the polymeric scaffolds.

Samples	Compression Strength (MPa)	Young’s Modulus (MPa)	Porosity (%)
BGH1	06.19 ± 1.3	35.73 ± 1.2	82.69 ± 1.1
BGH2	10.48 ± 1.1	56.07 ± 1.1	71.24 ± 1.2
BGH3	13.76 ± 1.1	113.75 ± 1.2	54.69 ± 1.2

**Table 4:** Summaries the swelling, biodegradation and wetting analysis.

Samples	Swelling (Aqueous) (%)	Swelling (PBS) (%)	Biodegradation (%)	Wetting 0 s (degree)	Wetting 10 s (degree)
BGH1	86.20 ± 1.3	71.90 ± 1.2	32.72 ± 1.1	112	106.10
BGH2	73.87 ± 1.1	58.40 ± 1.1	43.69 ± 1.2	92.40	89.20
BGH3	62.30 ± 1.1	47.87 ± 1.2	53.38 ± 1.2	81.40	74.70

## Data Availability

Data available within the article.
